# Diffuse unilateral subacute neuroretinitis: review article

**DOI:** 10.1186/s12348-019-0191-x

**Published:** 2019-12-27

**Authors:** Thiago José Muniz Machado Mazzeo, Mario Martins dos Santos Motta, André Luiz Land Curi

**Affiliations:** 1 Ophthalmology Residency Program, Federal University of the State of Rio de Janeiro (UNIRIO), Gaffrée and Guinle University Hospital, Rio de Janeiro, Brazil; 2 Ophthalmology Department, Federal University of the State of Rio de Janeiro (UNIRIO), Gaffrée and Guinle University Hospital, Rio de Janeiro, Brazil; 3 Research Laboratory of Infectious Diseases in Ophthalmology, National Institute of Infectious Diseases, Oswaldo Cruz Foundation, Rio de Janeiro, Brazil

**Keywords:** Eye infections, Uveitis, Choroiditis, Retinitis, Albendazole, Photocoagulation

## Abstract

Diffuse unilateral subacute neuroretinitis (DUSN) is an ocular infectious disease that can lead to severe visual impairment and blindness. It usually occurs in healthy young individuals and depending on the stage of the disease, it may present as vitritis, multifocal gray-white lesions in the outer retina, and derangement of the retinal pigment epithelium, narrowing of the retinal vessels and optic atrophy. Parasites of different sizes and species have been proposed as the etiologic agent of DUSN, including *Ancylostoma caninum*, *Toxocara canis*, and others. Thus, it is hypothesized that different infectious worms may be considered as the likely cause of both an autoimmune and toxic form of nematode retinopathy. Because serologic testing is variable, the definitive diagnosis is made when clinical characteristics of DUSN are found in conjunction with an intraocular worm. Ancillary tests can assist in the differential diagnosis when the nematode cannot be visualized, such as fluorescein and indocyanine green angiography, electrophysiological tests, visual field studies, and more recently, optical coherence tomography angiography. Cases in which the worm can be identified, it is defined as confirmed DUSN, and eyes with the typical clinical features but without identification of the worm should be classified as presumed DUSN. In confirmed DUSN, the classic treatment is directly photocoagulation of the worm; however, it can only be visualized in 30% (to 40%) of cases. Treatment of presumed DUSN cases with high-dose oral albendazole has shown encouraging results. However, perhaps due to the disease’s rarity or its underdiagnosis, there are no studies comparing current treatment modalities in both presumed and confirmed DUSN. Due to the possibility of this disease being, in part, autoimmune nematode retinopathy, corticosteroids associated with both albendazole or laser therapy, could be in any way beneficial. Thus, further comparative studies are necessary to elucidate the best treatment for this potentially blinding disease.

## Background

The objective of this article is to do a prospective literature review, describing the manifestations and etiology of DUSN, as well as its pathogenesis, diagnosis, and current types of management. The treatment is discussed according to the point of view of various authors, since, to the best of our knowledge, there is no consensus about the best treatment plan for both confirmed and presumed DUSN, which ranges from oral antihelmintic (albendazole), corticosteroids, to worm photocoagulation.

## Introduction

Diffuse unilateral subacute neuroretintitis (DUSN) is an ocular infectious disease that can lead to visual impairment and blindness. It was first described by Gass in 1978 [1], in which healthy young individuals presented with insidious, usually severe unilateral visual loss, vitritis, diffuse, and focal pigment epithelial derangement in the earlier stages of the disease. If left untreated and the inflammation persists, the disease can lead to narrowing of the retinal vessels and optic atrophy, causing permanent vision loss. Despite DUSN being mostly described as unilateral disease, rare bilateral cases are reported in the literature [1–6].

A worm, whose etiology has not been completely elucidated, is thought to be responsible for this inflammatory and degenerative process in neurosensory retina and retinal pigment epithelium (RPE). Infection of the eye by a nematode was first reported in the year 1950 by Wilder [7] in enucleated eye specimens, and Parsons [8] provided the first report of a subretinal mobile worm causing an ocular syndrome in 1952. One of the first reported Brazil cases was described in 199 [9–14]..

The subretinal worm is found in less than half of the cases, and most patients are under 20 years of age with already severe visual impairment, due to presenting in the later stages of the disease, where the likelihood of improvement is low, despite therapy. In cases of early diagnosis, prompt treatment, whether with oral anti-helminthic or direct photocoagulation of the worm, patients may show good visual improvement and have a more favorable prognosis [3, 15–18].

The objective of this article is to describe the manifestations and etiology of DUSN, as well as its pathogenesis, diagnosis, and current types of management.

## Etiology and epidemiology

Parasites of different sizes and several species of nematodes have been proposed as the etiologic agent of DUSN, including *Toxocara canis*, *Baylisascaris procyonis*, *Ancylostoma caninum*, and others. *B. procyonis* and *A. caninum* are thought to be the most common infectious agents, but despite many efforts, no conclusion has been reached about the specific organism causing this disease. Precise diagnosis of these nematodes is also limited due to the lack of good serologic testing or stool examinations, and most reports are made based on clinical exam. Thus, the majority of the studies argue that different agents may be considered as the likely cause of the same entity, called DUSN [1, 3, 6, 15, 19, 20].

Since DUSN is thought to be an ocular final common result of different infectious microorganisms, the mode of transmission will vary accordingly. In regions from the southeastern of the USA and South America, the nematode may vary in length from approximately 400 to 700 μm. In other areas, such as the north Midwestern USA, it measures approximately 1500 to 2000 μm in length. Moraes et al. reported the first South American case of DUSN caused by the larger nematode [3, 6, 21].

Previous reports tried to identify the nematode, even through the retinal biopsy (transscleral approach); however, due to poor histologic details, precise identification of the nematode was not possible. Agent identification in the majority of studies is based on a combination of measuring the parasite, serologic testing, and epidemiological data, all of which have limited accuracy [5, 9, 22].

Oréfice et al. [23] found a 47.62% positivity for enzyme-linked immunosorbent assay (ELISA) in a case series of 23 DUSN patients, and in 2 with the live nematode, the ELISA was negative. Other studies observed that ELISA anti-*T. canis* was negative in most of the 39 DUSN cases he studied [19].

### T. canis

Gass and Olsen [9] previously suggested that *T. canis* was not the causative microorganism due to the lack of serologic evidence. In addition, the clinical picture was unlike that associated with ocular toxocariasis, but Oppenheim et al. reported a case of *Toxocara* DUSN in which the patient’s positive ELISA titer decreased fourfold over a 2-year period. This suggests that the serologic variability may be a reflection of the timing of the serology in relation to the onset of the disease or the immune status of the patient [24].

### B. procyonis

Significant morphometric, serologic, and epidemiologic support for *Baylisascaris* as the causative agent of DUSN in northern climates was published by Goldberg et al. [25]. It is described to be a large nematode (ranging from 400 to 2000 μm) found in raccoons and may cause not only DUSN in patients, when infectious eggs from feces are ingested, but also visceral-ocular larva migrans and eosinophilic meningoencephalitis. Even though most patients have no history of exposure to raccoons, large nematode DUSN patients are seen to be from areas of the USA where raccoons are not only common but also infected with *B. procyonis* [5, 12, 26].

### A. caninum

It is a common cause of dog parasitic infection in South America that cause cutaneous larva migrans (CLM) in humans when infectious eggs from dog feces are ingested or when the larvae enter through the skin (usually the foot). It is also a frequent cause of CLM in the southeastern USA, and occasionally, it may precede the onset of DUSN [1, 3, 17].

It is described to be one of the small nematodes (approximately 650 μm) that cause the syndrome. The infective larva of *A. caninum* is capable of surviving in host tissue, for months and probably years without changing size or shape. No serologic test is currently available for *Ancylostoma* [9, 27].

## Pathophysiology

The pathogenesis of DUSN may be related to the presence of a larva in subretinal space, promoting an extensive inflammatory and degenerative process of both the retina and retinal pigment epithelium (RPE). Infiltration of eosinophils, macrophages aggregates, and gliosis may affect all retinal layers, leading to variable loss of ganglion cells. Toxic products released by the larva in the subretinal space would locally affect the external portion of the retina, and a diffuse tissue reaction would lead to external and internal retinal damage. Vascular narrowing and progressive ganglion cell loss occur until optic atrophy establishes [9].

So that, the association of worm’s migration through the neurosensorial retina, the toxicity of its excretions and the host immunological response are thought to be the main pathophysiological mechanisms, converging into a toxic autoimmune nematode retinopathy [3, 28].

## Clinical characteristics

DUSN is most frequently seen in healthy children or young adults with no significant past ocular history. The clinical findings depend on the stage of the disease, where it can be described as early or late and can vary from evanescent multifocal white-yellowish lesions at the level of the outer retina and choroid, to narrowing of the retinal vessels and optic atrophy [3, 9, 29].

### Early stage

**Fig. 1:**
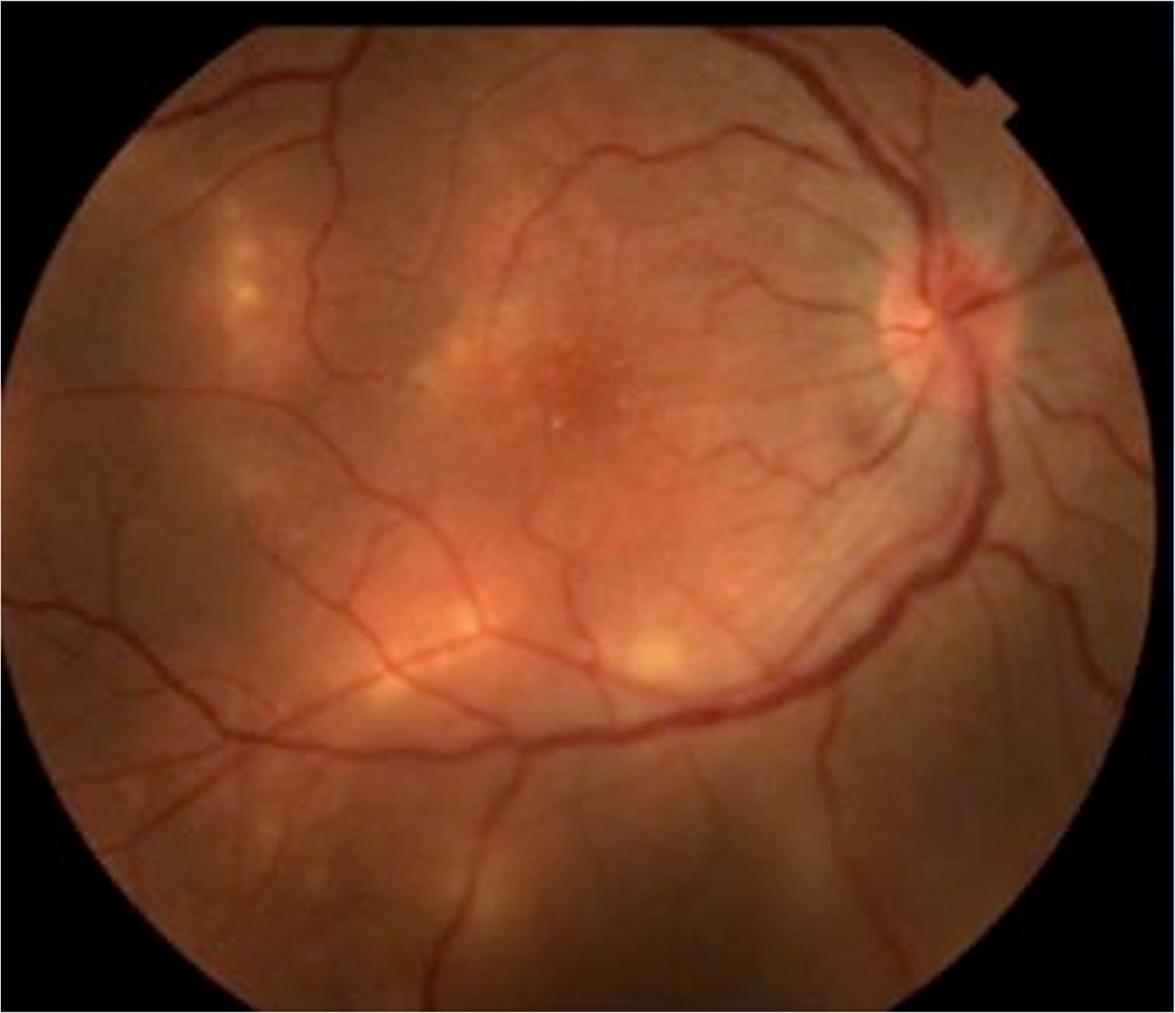
Multifocal gray-white lesions in the right eye of a presumed DUSN patient.

In the acute phase of the disease, patients usually present visual loss that is often related to vitritis and optic disc edema. Evanescent multifocal gray-white lesions (Fig. 1) at the level of the outer retina, which typically cluster in one segment of the fundus, can also be visualized. These lesions are thought to be related to possible reactions to the nematode in subretinal space and characteristically migrate and change according to worm’s location. It may or may not leave a residual retinal lesion depending on the degree of host immune response against the toxic products of the worm [20, 30–32].

As the worm moves to another part of the retina, the lesions fade, explaining the evanescent aspect of these gray-white lesions and its reappearance in a different place, whether in the vicinity of the previous ones or not. Visual loss is rarely reversible and is usually worse than 20/200 in approximately half of the patients. Central or paracentral scotomas in the visual field may also be present [1, 20].

In approximately 25 to 40% of cases, a worm is visualized during the eye examination.

The intraocular worm is seen as a motile, white nematode that varies in length from 400 to 2000 μm. Some worm species are more photosensitive than others, so that, examining light from slit lamp may cause the worm to move, a behavior which may be especially useful in cases of worms located in the macula, stimulating them to move away to an area where photocoagulation of the worm can be safely performed [29, 31, 33, 34].

Gass and Braunstein [20] reported that there is a greater likelihood of the longer worm leaving a tract of coarse clumping of RPE in the wake of its travels, whereas the shorter worm tends to leave focal, chorioretinal atrophic scars. The focal pigment epithelial changes are related to the travel pattern of the worm, while focal chorioretinal white spots are induced by an immune response to secretion or excretion from the worm. Diffuse pigment epithelial changes are suggested by some authors, to be related to a toxic reaction. Less frequent findings include iridocyclitis, subretinal hemorrhages, perivenous exudation, macular cysts, local retinal detachments, and subretinal neovascularization [1, 5, 16].

### Late stage

**Fig. 2:**
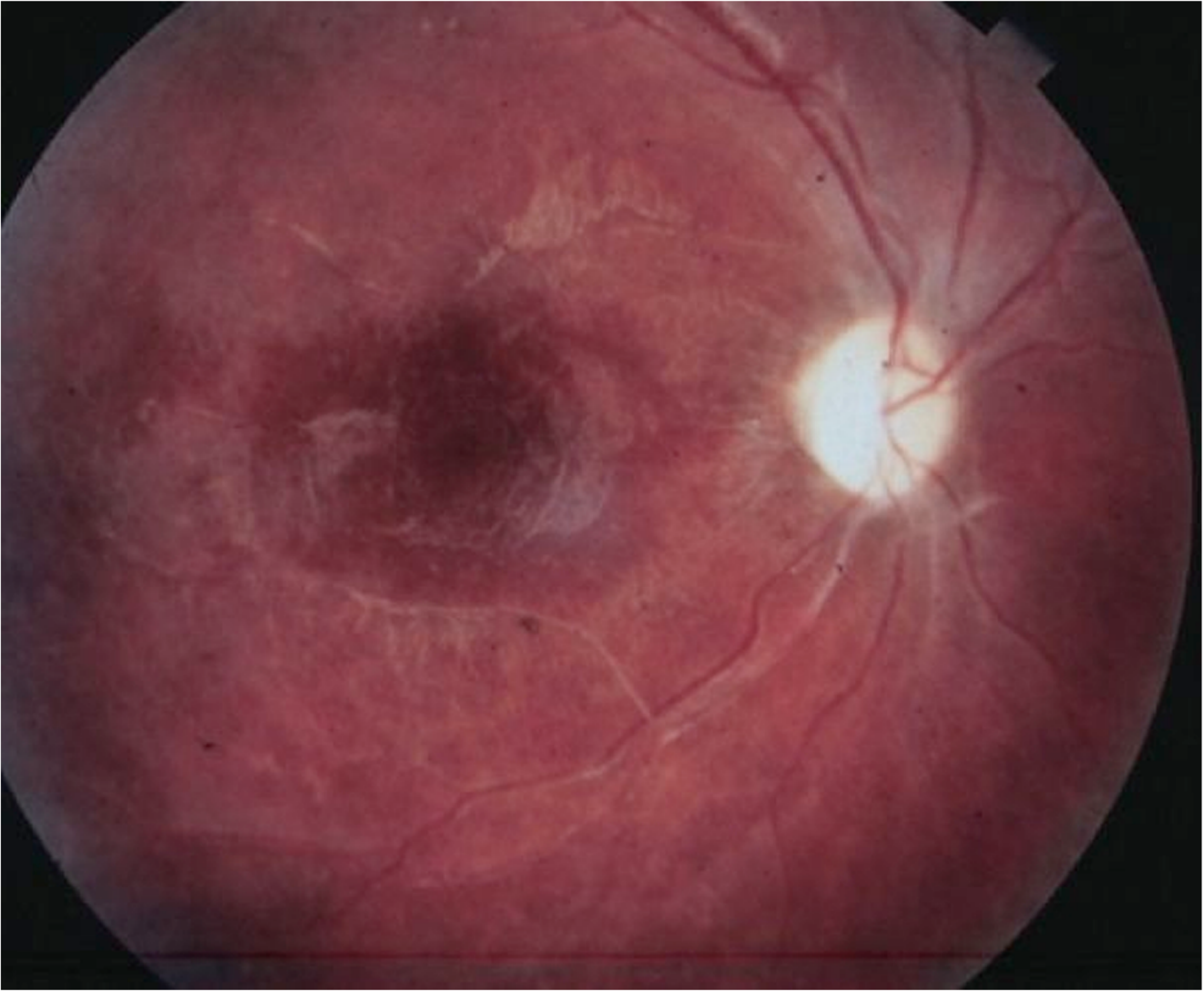
In the later stage of DUSN, patient fundus presented with a pale optic disc, narrowed retinal vessels, and pigmentary alterations

In the later stages of the disease, the patient may present mild vitritis, evidence of tunnels by optical coherence tomography (OCT) in the subretinal space (Garcia’s sign), focal, and diffuse degenerative changes in the RPE and retina. Vascular narrowing and progressive ganglion cell loss occur until the optic disc becomes atrophic (Fig. 2), leading to permanent vision loss [2, 3, 31, 35].

Retinal arteriole narrowing may vary by quadrant, and in conjunction with optic atrophy, it usually accompanies progressive changes in the RPE. According to some authors, toxic products released by the larva in the subretinal space would locally affect the external portion of the retina, whereas a more diffuse tissue reaction would damage all retinal layers [2–4, 9, 28, 30].

Visual acuity can be profoundly impaired, with 80% or more of the patients showing 20/200 or worse. Over weeks or months, diffuse and focal depigmentation of the RPE occurs, usually most prominent in the peripapillary and peripheral retina and less prominent in the central macular area [15, 16, 31, 36].

## Ancillary testing

### Fluorescein angiography

There is hypofluorescence of the focal gray-white lesions of active retinitis followed by staining, in the early stage of FA. Dye leakage can be observed from the capillaries on the optic disc, and occasionally, in a perivenous pattern. In the later stages of the disease, FA shows considerable pigment loss from the RPE, manifesting as an irregular increase in the background choroidal fluorescence, characterizing a “window defect” [9].

### Indocyanine green angiography

The choroid also seems to be involved in early-stage DUSN. Hypofluorescent dark spots seen in the affected eye are probably due to choroidal infiltration, which prevents normal indocyanine green angiography (ICG-A) impregnation. These dark spots, present in the initial ICG angiography phase, seem to either disappear or persist in the late phase of the examination. Hypofluorescent dots persisting in the late phase are interpreted as full-thickness lesions allowing no ICG diffusion, whereas dots becoming isofluorescent through the exam phases are interpreted as partial-thickness lesions progressively surrounded by the dye of adjacent tissues [37].

### Electroretinogram and electro-oculogram

DUSN may present a negative electroretinogram (ERG), which can also be found in ischemic retinal diseases, indicating significant dysfunction affecting the inner retina. This characteristically pattern shows a flat and below the normal response of B wave and a decrease in relation to B/A waves. Electroretinographic changes also include a mild to moderate decrease in rod and cone function, in which the B wave is usually more affected than the A wave [3, 30].

The mechanism of this finding can be explained as being a consequence of a possible autoimmune, inflammatory, and/or toxic aggression toward retinal bipolar cells. The ERG in the affected eye is usually abnormal even in the early in the course of the disease. About 50% of these patients can have a normal electro-oculogram (EOG), and the finding of normal EOG and abnormal ERG suggests a neuroepithelium disease. It is important that the ERG is rarely extinguished completely, which differentiates it from some tapeto-retinal degeneration [19, 35, 38].

Multifocal ERG findings before laser treatment showed decreased foveal response density and increased parafoveal and perifoveal waveform amplitudes. Two months after the laser photocoagulation of a subretinal nematode, multifocal ERG showed full recovery and visual acuity remained 20/20 [39].

### Visual field studies

Visual fields show different lesion patterns that cannot be correlated with the findings of the ocular fundus changes. However, Goldmann perimetry is useful to evaluate the remaining visual field before and after disease’s treatment [18, 34, 40].

In DUSN, visual field improvement following photocoagulation of the worm has been described, but the exact mechanism is not yet fully understood. It is hypothesized that the toxic inflammatory reaction induced by the worm is reduced over the retinal pigment epithelium and adjacent tissues, after nematode eradication [4, 20, 27, 33, 41, 42].

### Optical coherence tomography

Optical coherence tomography (OCT) can be important for the characterization of the clinical picture of DUSN, given that the worm cannot always be found, and therefore, it may help to identify the disease, assisting in the differential diagnosis and also monitor treatment’s anatomical response [30, 43].

OCT common findings are neuroretinal atrophy, focal hyper-reflectivity through the retina in areas affected by the worm, and general loss of the inner retinal layer. However, progressive changes can range from the retinal nerve fiber layer (RNFL) to the RPE, since the worm and its toxic effects can affect all retinal layers, not only the subretinal space as it was first described [14, 30, 36].

Studies observed that there was no significant difference between the RNFL thickness in patients with or without a live worm. Despite this, it is possible to have both increase (due to transitory edema) and decrease (secondary to nerve fiber loss) of RNFL thickness, as the disease progresses. In addition, decreased RNFL thickness has been previously correlated with worse visual acuity [35, 44].

### Optical coherence tomography angiography

Gass initially described that high-quality color fundus photos were the best way to identify nematodes. However, with the worm not been found in over 50% of suspected DUSN cases, there is still significant room for other techniques. Studies demonstrated that optical coherence tomography angiography (OCT-A) is another imaging modality that can assist in the diagnose. The nematode can be detected as long as it moves because nematodes have no vascular system. However, it is possible that an inactive worm may not be detected [1, 45].

## Diagnosis

When a worm is identified within the eye of an otherwise healthy person, unless there is peripheral eosinophilia, no further evaluation seems warranted to make the diagnosis. The nematode can be visualized in both acute and chronic phases of the disease, and in cases in which it can be identified, it should be defined as confirmed DUSN, and eyes with the typical clinical features but without identification of the worm should be classified as presumed DUSN. De Amorim Garcia Filho et al. described in a large series of 121 patients that the subretinal word could only be identified in approximately 40% of the patients [6, 16].

So that, despite DUSN being sometimes difficult to diagnose, especially in early stages and when the worm cannot be visualized, it should always be suspected in healthy patients with unilateral insidious vision loss, retinal vasculitis, multifocal lesions involving deep retinal layers, narrowing of the retinal vessels, or optic atrophy. The ancillary tests can both assist in the differential diagnosis and monitor the disease’s progression [17, 41].

## Differential diagnosis

In earlier stages of the disease, DUSN is often mistaken for other entities, including toxoplasmosis, toxocariasis, histoplasmosis, multifocal choroiditis, serpiginous choroiditis, acute posterior multifocal placoid pigment epitheliopathy, multiple evanescent white dot syndrome, and optic neuritis [17]

In later stages, it is important to exclude posttraumatic chorioretinopathy, unilateral retinitis pigmentosa, occlusive vascular disease, sarcoidosis, syphilis, and other toxic retinopathies [9, 32].

## Treatment

### Laser treatment

Classically, laser photocoagulation is the only recommended treatment for DUSN once the causal nematode has been located; however, searching and photocoagulation of the worm can be a very laborious and time-consuming task, requiring a great deal of experience**.** Despite this, laser treatment seems to offer a good chance for halting worm’s movement, decreasing intraocular inflammation and toxic damage to eye tissues. For this reason, some improvement in vision and visual field may occur after laser treatment of the worm; however, in late stages of the disease, laser treatment may not be so effective [21, 27, 34, 40, 46].

In some patients where the worm is close to the macula and heavy photocoagulation may damage the fovea, it may be possible to use low level illumination or light applications of the laser to chase the worm into the mid periphery, due to its photosensitivity, where it may be destroyed with less risk of compromising patient’s central vision. In addition, laser treatment should be performed as soon as the worm is identified, because they are motile and may be difficult to locate it if the procedure is delayed. Clinical treatment is limited to cases in which no worm is found despite repeated examinations [19, 34, 47, 48].

### Oral treatment

The search for an oral treatment for DUSN started in 1980 with Gass and associates. After several trials with oral antihelmintic drugs, such as thiabendazole and diethylcarbamazine, it was found that the tested drugs would only destroy the subretinal worms in some of their patients. Thereby, it was hypothesized that these antihelmintic drugs would depend on an inflamed eye (blood-retinal barrier breakdown), justifying the variable efficacy [20, 49–52].

In such a way, thiabendazole and corticosteroids only demonstrated efficacy in cases where the worm cannot be found and with a moderate degree of vitreous inflammation, indicating a breakdown in the blood-retinal barrier (BRB). In this same group of patients, Gass and Olsen previously proposed the use of moderately intense scatter photocoagulation in the vicinity of the white lesions to induce the breakdown of the BRB before the administration of thiabendazole, in order to maximize its efficacy [18, 53].

Later on, in the search for a more effective drug for DUSN capable of crossing an intact blood-retinal barrier, experiments were done with oral albendazole, since it is an effective broad-spectrum antihelmintic drug capable of crossing the intact blood-brain barrier and has not been associated with marked systemic side effects. It acts by binding to parasite beta-tubulin, inhibiting its polymerization and impairing glucose uptake. Single-dose albendazole (400 mg) is believed to achieve cure rates of 90% against *Enterobius vermicularis*, *Ascaris lumbricoides*, hookworm, cutaneous larva migrans, and giardiasis [42, 46, 54, 55].

Souza et al. reported the drug efficacy in the first case resulting in a dramatic visual improvement from counting fingers to 20/30 after 1 month of treatment. In the same report, he also described 12 Brazilian patients who had significant improvement of visual acuity, visual field, and ocular inflammation after exclusive treatment with high-dose oral albendazole (400 mg/d) for 30 days. During the first weeks of treatment, it was observed worm inactivation in four patients in whom the worms were visible and no adverse drug side effects were related [18].

The optimal dosing and duration of treatment for DUSN with albendazole have still not been determined, but the suggestion to use it for 30 consecutive days was based on the good results observed in patients with neurocysticercosis. The oral absorption is low and needs to be enhanced by fatty meals. Side effects are reported as being few and mild, such as gastrointestinal upset, dizziness, rash, and alopecia. Improvements in central visual acuity (at least two lines of Snellen) and visual field (central scotoma reduction and peripheral field enlargement) were registered in the majority of patients [4, 22, 33, 42, 46, 54, 55].

## Conclusion

Regardless of the nature of the causative organism, and if it the nematode can be visualized or not, DUSN should always be suspected in healthy patients with unilateral insidious loss of vision, vitreous inflammation, retinal vasculitis, multifocal lesions involving deep retinal layers, narrowing of the retinal vessels, or optic atrophy.

Laser photocoagulation offers the best chance for clinical resolution of the disease, but the worm is visualized in only a small portion of the cases, in which other treatments such as high-dose albendazole have been used with efficacy and safety; however, the optimal treatments’ dose and duration has still not been determined. Despite this, it is very important to establish the diagnosis in the early phase when worm photocoagulation or oral antihelmintic has the greatest likelihood of vision improvement.

In the literature, there are no studies comparing treatment’s regimen in both presumed and confirmed DUSN. Due to the possibility of this disease having an immunological element, corticosteroids associated with both high-dose albendazole and laser therapy could be in any way beneficial. Thus, further studies are necessary to better elucidate the efficacy of current treatment modalities and its associations depending on the stage of this potentially blinding disease.

## Data Availability

presented in the main paper (references) or additional supporting files

## Abbreviations

DUSN: Diffuse unilateral subacute neuroretinitis
RPE: Retinal pigment epithelium
OCT: Optical coherence tomography
FA: Fluorescein angiography
ERG: Electroretinogram
ELISA: Enzyme-linked immunosorbent assay
ICG-A: Indocyanine green angiography
EOG: Electro-oculogram
RNFL: Retinal nerve fiber layer
OCT-A: Optical coherence tomography angiography
BRB: Blood-retinal barrier
